# Consciousness: New Concepts and Neural Networks

**DOI:** 10.3389/fncel.2019.00302

**Published:** 2019-07-09

**Authors:** Tong Zhao, Yiqian Zhu, Hailiang Tang, Rong Xie, Jianhong Zhu, John H. Zhang

**Affiliations:** ^1^Department of Neurosurgery, Huashan Hospital, State Key Laboratory of Medical Neurobiology, Institutes of Brain Science, Collaborative Innovation Center for Brain Science, Shanghai Medical College, Fudan University, Shanghai, China; ^2^Center for Neuroscience Research, Loma Linda University School of Medicine, Loma Linda, CA, United States

**Keywords:** consciousness, neural network, vegetative state, plasticity, brain injury

## Abstract

The definition of consciousness remains a difficult issue that requires urgent understanding and resolution. Currently, consciousness research is an intensely focused area of neuroscience. However, to establish a greater understanding of the concept of consciousness, more detailed, intrinsic neurobiological research is needed. Additionally, an accurate assessment of the level of consciousness may strengthen our awareness of this concept and provide new ideas for patients undergoing clinical treatment of consciousness disorders. In addition, research efforts that help elucidate the concept of consciousness have important scientific and clinical significance. This review presents the latest progress in consciousness research and proposes our assumptions with regard to the network of consciousness.

## Introduction

Neuroscientists have conducted extensive research on consciousness for many years. In the past, the traditional viewpoint was that consciousness required the proper functioning of midline brain structures and that the content of an experience was supported by the activity of neurons in specific areas of the cerebral cortex (Jang and Lee, 2015). The reticular neurons, and especially the neurons of the ascending reticular activation system, play a vital role in maintaining behavioral arousal and consciousness (Jones, 2003; Englo et al., 2017). However, in recent times, many different opinions have been proposed. For example, some researchers believe that consciousness is aroused in the frontal region of the brain, including the prefrontal and central anterior cortex. Others believe that consciousness is created in areas of the hindbrain, including the occipital/parietal and central posterior regions of the brain (Koch et al., 2016; Seth, 2018). Questions that should be asked include: where is the material basis of consciousness? The physical basis of consciousness is the most important internal factor of consciousness.

## Important Component of Consciousness: Wakefulness

**FIGURE 1:**
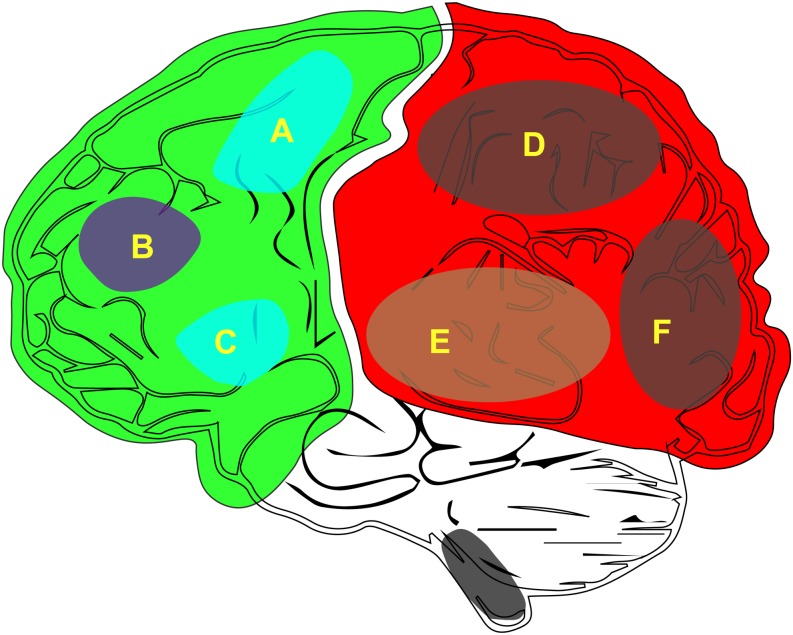
The distribution of the neurobiological basis of consciousness in the brain. **(A)** M1, primary motor cortex. **(B)** Attention or working memory. **(C)** Verbal report (Broca). **(D)** Other content of consciousness. **(E)** Auditory consciousness. **(F)** Visual consciousness.

According to the latest neurosurgical research there are two key features of consciousness: (1) the state of consciousness (i.e., wakefulness) and (2) the content of consciousness (i.e., awareness) (Zeman, 2006; Bayne et al., 2016; Fazekas and Overgaard, 2016). Furthermore, there are additional features of the content of consciousness across the context of our brain (Figure 1). From the perspective of neurosurgeons, more attention is paid on wakefulness than on awareness because a disorder of this state can lead to coma. In the clinic, comas, and the associated vegetative state, are difficult to resolve (van Erp et al., 2015; Baricich et al., 2017).

Consciousness includes both the level of consciousness and its content. The level of consciousness is key to maintaining sobriety (Bayne et al., 2016). The generation of consciousness content (Figure 1) depends on the integration of the various sensations in the posterior cortex of the brain. Being able to stay awake and aware of the outside world is a characteristic that we associate with a conscious person (Fazekas and Overgaard, 2016). In addition, to perceive the outside world, one must first keep a clear head; that is, waking is the first step in the generation of consciousness. Therefore, if one wishes to study the generation of consciousness, one can start with wakefulness. Furthermore, the opposite of awakening is coma, which is helpful for the study of the mechanisms associated with clinical coma. Wakefulness research is relatively easy when compared to the study of the content of conscious because, unlike awareness, wakefulness can be quantified and is easier to observe and record.

As a state of consciousness, wakefulness is different in the context of both sleeping and coma. These states are mild or severe. For instance, one can wake up or semi-wake up, just as one could have mild or deep anesthesia. We are usually confident in judging a person’s state of consciousness (Zeman, 2006). In the first sense, with the help of objective criteria, including the Glasgow Coma Scale (GCS), opening of the eyes usually indicates the state of being awake, and being able to talk almost always indicates further wakefulness. It is often thought that an awake individual is also aware; however, this is not always true. Actually, most of the content of consciousness is based on the state of awakening, and brain activity during sleep is a special case.

Research on consciousness has always been difficult. For example, the definition of consciousness is very vague and includes many aspects, such as the disciplines of psychology and philosophy. Thus, there is no definitive conclusion. In addition, the assessment of the level of consciousness is not an easy task. Awakening is an important component of consciousness and is also a well measured state by the availability of existing means. Therefore, the scientific exploration of consciousness should begin with research that is focused on the state of awakening. This information may further reveal the mysteries of consciousness.

## Key Nuclear Events Related to Consciousness: The Paraventricular Nucleus and Claustrum

### Paraventricular

The paraventricular nucleus is an important neurosecretory nucleus of the hypothalamus. Located in the medial area of the hypothalamus, above the upper nucleus, the paraventricular nucleus transmits from the pituitary gland to the posterior pituitary (Maejima et al., 2017; Qin et al., 2018). Previous studies have suggested that the paraventricular nucleus is an endocrine nucleus that produces a variety of hormones, including antidiuretic hormone and oxytocin. Further, scientists have revealed that there is an association between the paraventricular nucleus and hunger, appetite, drug addiction, and behavioral control (Krashes et al., 2014; Kirouac, 2015; Millan et al., 2017). However, the paraventricular nucleus also promotes sleep awakening and increases arousal (Kirouac, 2015). Orexin acts upon orexin receptors and are involved in the regulation of sleep-wakefulness (Ohno and Sakurai, 2008). Orexin neurons around the lateral fornix of the hypothalamus are mainly projected to the paraventricular nucleus of the thalamus, which is deeply involved in the control of motivated behaviors. Orexin-activated paraventricular thalamus (PVT) neurons play roles in the integration of sleep-wakefulness (Ishibashi et al., 2005). Therefore, the paraventricular nucleus of the thalamus may be the key site involved in the control of arousal. This is consistent with the view that the PVT and thalamic midline nuclei are associated with important physiological mechanisms, such as attention, arousal, and consciousness (Groenewegen and Berendse, 1994; Nascimento et al., 2008). Moreover, the PVT is a member of the midline and intralaminar group of thalamic nuclei that were originally hypothesized to function as the thalamocortical arousal system (Jones, 2003) and transmit information related to the state of awakening (Van der Werf et al., 2002).

Clinical observations have indicated that the central region of the thalamus is a key node for controlling the state of awakening; however, the key nuclei or neural circuits of this functional region remain unknown. By using *in vivo* fiber photometry and multi-channel electrophysiological recordings in mice, Dr. Hu (Ren et al., 2018) found that glutamatergic neurons of the PVT showed high activity during waking, and inhibition of PVT neuronal activity led to decreased arousal. Additionally, activation of PVT neurons induced a transition from sleep to arousal and from a waking-up process after accelerated general anesthesia. This study also demonstrated that the projection of the PVT nucleus accumbens and the projection of orexin-secreting neurons from the lateral hypothalamus to the PVT glutamatergic neurons represent the pathways that control arousal. These results indicated that the PVT is the key thalamic nucleus that controls arousal.

### Claustrum

The claustrum is a thin, irregular sheet of neurons that is attached to the underside of the neocortex in the center of brain. It is suspected to be present in the brains of all mammals. Although it is known that the claustrum and the cerebral cortex are reciprocally connected, little is known about the actual function of the claustrum. The claustrum and the frontal lobe are interconnected, and it includes the motor cortex, prefrontal cortex, and cingulate cortex; it also includes the occipital lobe of the visual cortex, the temporal and temporal cortex, the occipital and posterior parietal cortex, the frontal cap, the somatosensory area, and the anterior axillary olfactory and entorhinal cortex (Gattass et al., 2014; Goll et al., 2015; Torgerson et al., 2015; Dehaene et al., 2017; Kitanishi and Matsuo, 2017; White et al., 2018), which also projects into the hippocampus, amygdala and the caudate nucleus (Milardi et al., 2015). The claustrum may be closely related to the generation of consciousness since it provides functional anatomical links between the frontal cortices and the posterior sensory/association cortices (White and Mathur, 2018). The extensive reciprocal connections of the claustrum with almost the entire neocortex prompted us to propose its role as a gateway for perceptual information to the arousal system.

Koubeissi et al. (2014) reported that they were able to control a woman’s consciousness by stimulating the claustrum. The woman in this study was epileptic; thus, the researchers used electrodes implanted deep in her brain to record signals from different brain regions during seizures in an attempt to cure her. One of the electrodes was next to the claustrum, and when they stimulated this area with a high-frequency current, the woman lost consciousness. Additionally, she stopped reading, showed “fragments” blankly, breathed slowly, and was unresponsive to the audience and visual instructions. When the stimulus stopped, she immediately regained consciousness and was completely unaware of what had happened. The same situation occurred several times during the test (Koubeissi et al., 2014). Another experiment in 2015 also supported the fact that claustrum may be associated with consciousness. The scientists in this study examined 171 veterans with traumatic brain injuries and observed the effects of claustrum damage on consciousness. They found that claustrum damage was associated with the duration of the loss of consciousness, but not with the frequency. Moreover, they believed that the claustrum played an important role in the restoration of consciousness but that it had little to do with the maintenance of consciousness (Chau et al., 2015).

Based on previous research, we assumed that consciousness was generated by the claustrum, which acted as a command center and formed a network covering the entire brain through the interconnection of three types of neurons, including neurons in the prefrontal lobe, the posterior frontal sulcus and the occipital lobe region. We also assumed that the prefrontal lobe and the state of high-level consciousness (cognition) were functionally related. In addition, we believed that the most basic form of consciousness may be generated by the posterior and occipital regions of the central sulcus. Although the claustrum is closely related to the cerebral cortex and various important nuclei, its connection is different. Most of the projection fibers of the cerebral cortex and nucleus that connect with the claustrum are derived from the bilateral brain, and the nerve fibers emitted from the claustrum are mostly directed to one side. In other words, the projection of the claustrum to the outside is asymmetrical (Smith et al., 2017). Damage to the claustrum of each cerebral hemisphere may also show some differences, although these differences still need to be confirmed (Zingg et al., 2014).

## Vital Cortical Consciousness Regions: The Hindbrain and Posterior Cortical Region

By using functional magnetic resonance imaging (MRI), we are able to observe when the brain is unable to stimulate, when corresponding regions are activated, and when neurons become abnormally active. These differences were used in word stimulation and visual decision task experiments, and it was concluded that activation of the cerebral hemisphere depends on the nature of the task rather than the stimulus itself. Whether activated on the left or the right side, activation of the brain is not in the prefrontal cortex. Rather, activation is observed in the vicinity of the central sulcus and the back of the brain (Stephan et al., 2003). In patients who underwent post-traumatic surgery to remove some of brain regions, the vast majority of patients (98%) remained in a persistent vegetative state after one year if the resected section involved the posterior cortex (Boly et al., 2017). Bianchi’s research also reached a similar conclusion, and believed that lesions in the posterior cortex of the brain may lead to permanent coma (Bianchi and Sims, 2008). Neurological awareness is primarily anatomically located in the posterior cortical thermal region, including the sensory region, rather than the prefrontal network that is involved in task monitoring and reporting (Merker, 2007). Reports of patients that remain conscious after bilateral frontal lobe resection indicate that the prefrontal cortex is not essential for consciousness (Rowland and Mettler, 1949). Other parts of the cerebral hemisphere may be potential candidates for the maintenance of consciousness, including the back part of the brain.

## Crucial Theories of Consciousness: Global Workspace Theory, Integrated Information Theory, and Quantum Theory

The most effective way to solve problems associated with consciousness is to use descriptions that have been introduced by psychologists and cognitive scientists who strive to connect different aspects of their models to the neuroanatomy and neurophysiology of the brain (Harris and Shepherd, 2015). In the history of consciousness research, several theories have attempted to conceptually explain consciousness by presenting neural correlations of the stream of consciousness. In the following sections, we will discuss three of the most popular theories.

### Global Workspace Theory

The global workspace (GW) theory of consciousness was first proposed by Baars in 1988 (Baars, 2005) and developed by Dehaene et al. (1998). The GW theory is based on competition, and its core idea is that conscious cognitive content can be used globally for a variety of cognitive processes, including attention, assessment, memory, and verbal reporting. According to the GW theory, a single brain region cannot independently accomplish the task of generating consciousness. Instead, consciousness requires the joint participation and coordination of different parts of the cerebrum. This encourages us to not limit the concept of consciousness to a single brain functional area or a star nucleus and to explore the brain as a whole.

The GW theory posits that computers of the future will be conscious (Thagard and Stewart, 2014; Dehaene et al., 2017). There are some disadvantages of the GW theory. First, it provides, at best, an account of the cognitive function of consciousness but fails to address the deeper problem of the nature of consciousness (i.e., what consciousness is) and how any mental process can be conscious (i.e., the “hard problem of consciousness” hypothesis) (Thagard and Stewart, 2014; LeDoux and Brown, 2017). Second, “while this hypothesis does not address the ‘hard problem,’ namely, the very nature of consciousness, it constrains a theory that attempts to do so, and provides important insights into the relationship between consciousness and cognition” (Northoff and Huang, 2017).

### Integrated Information Theory

The essence of the integrated information theory (IIT) is that consciousness is the capacity of a system to integrate information. It is the most audacious current proposal of Giulio Tononi’s hypothesis (Tononi, 2004). Instead of focusing on the function of the cerebrum the IIT starts from the results in an attempt to determine the reason. Moreover, the IIT presents a mathematical framework for evaluating the quality and quantity of consciousness (Tononi, 2012; Oizumi et al., 2014; Tononi et al., 2016; Tsuchiya et al., 2016). This theory posits that the physical basis of consciousness must be the maximization of internal causal power and provide a means to determine the quality and quantity of an experience (Tononi, 2004). The theory leads to some counterintuitive predictions and can be used to develop new tools for assessing consciousness in non-communicative patients. However, the IIT proposes conditions that are necessary for consciousness but that are not entirely sufficient (Nathan and Barbosa, 2011). The IIT claims that all of its axioms are self-evident (Kung et al., 2019). Since the IIT is not a functionalist theory of consciousness, criticisms of non-functionalism have been levied against it (Kung et al., 2019), and the limits of the IIT’s definition of consciousness have led to criticism (Nathan and Barbosa, 2011; Kung et al., 2019).

### Quantum Theory

It is well known that consciousness is inextricably linked to anesthesia. Hameroff et al. (2002) have conducted many complementary and engaging studies to refine their quantum theory of consciousness. Moreover, a series of mechanics of anesthetic agent function were proposed and included the following: (1) selective binding in hydrophobic pockets comprising non-polar amino acid groups in brain proteins [e.g., microtubule-associated proteins (MAPs); tubulin; and van der Waals (London dispersion) forces, which can inhibit the quantum process by impairing electron mobility]; (2) a specialized combination with “qubits,” fundamental information units that abide by quantum events that induce disruption of the quantum computation; (3) a concept of “quantum channels” that consist of tryptophan tings in tubulin that are olive-like, non-polar, and hydrophobic; (4) probability for π-electron resonant energy transfer through quantum channels and weakening of anesthetic agents that could therefore weaken this π-resonance energy transfer (a theorem that accounts for loss of consciousness); and (5) alterations of collective terahertz dipole oscillations in tubulins (Hameroff, 1998, 2006; Hameroff et al., 2002; Craddock et al., 2015, 2017; Mayner et al., 2018). The systematic theory of “Orch OR” proposed that consciousness is constituted by discrete events that correspond with varying oscillation frequencies of distinct brain regions (Crick and Koch, 2003; Hameroff and Penrose, 2014; Li et al., 2018), which is similar with respect to the “snapshots,” which is one of the ten frameworks for consciousness proposed by Koch (Li et al., 2018). In addition, an “integrate-and-fire” brain neuronal model and three time-steps of a microtubule automaton have emerged from the above studies that embody specific processing in neuronal microtubules when consciousness has occurred. It seems increasingly feasible to explain consciousness on the basis of quantum mechanics because Li et al. (2018) demonstrated that an isotope of the anesthetic xenon (129Xe) displayed half the quantum property of nuclear spin and was conspicuously less potent than xenon isotopes lacking spin, despite no observed differences in terms of chemical reactions (Craddock et al., 2015, 2017).

Since the enigmatic riddle of consciousness is so intractable, we need additional theorems and hypotheses to be generated with the intent of increasing the level of attention. Perhaps this quantum theory will fade with elapsing time and gradually lose its “charm;” however, it is also possible to disperse the fog of our ignorance and shed light on a new level of comprehending consciousness and adopt a system of “wait and see.”

## Hypotheses: Fundamental Huge Neural Network of Consciousness

From the above literature, we have identified three key areas associated with the generation of consciousness, including the PVT (Groenewegen and Berendse, 1994; Van der Werf et al., 2002; Ishibashi et al., 2005; Nascimento et al., 2008; Ohno and Sakurai, 2008; Kirouac, 2015), the claustrum (Gattass et al., 2014; Koubeissi et al., 2014; Chau et al., 2015; Goll et al., 2015; Milardi et al., 2015; Torgerson et al., 2015; Dehaene et al., 2017; Kitanishi and Matsuo, 2017; White and Mathur, 2018; White et al., 2018), and the posterior cortex (the occipital and temporal lobe) (Rowland and Mettler, 1949; Stephan et al., 2003; Merker, 2007; Bianchi and Sims, 2008; Koch et al., 2016; Boly et al., 2017). Recent experimental studies have confirmed that the PVT plays a key role in animal arousal and that the animal’s arousal state can be regulated by the PVT (Hameroff, 2012; Kirouac, 2015). In patients with epilepsy, the state of consciousness can be reversed by stimulating the claustrum. Stimulation of the claustrum can cause loss of consciousness and stop epilepsy (Koubeissi et al., 2014). In addition, modern neuroscience research has suggested that consciousness includes both awakening and consciousness content. The generation of consciousness is from the awakening to the transformation of the content of consciousness (Tononi and Koch, 2015). Any problem that is associated with any one of the links could lead to the decline of the level of consciousness or even coma. The generation of consciousness depends on a neurobiological basis. The neural mechanism that produces the least consciousness is called the related neuron of consciousness (NCC) (Crick and Koch, 2005; Cerullo, 2015; Tononi and Koch, 2015; Reddy and Pereira, 2017; Xie et al., 2017), which was first proposed by Crick and Koch (1990). The study of the NCC is a key step toward research of consciousness. The NCC are distributed in all parts of the brain (Koch et al., 2016; Boly et al., 2017) and rely on neural networks or loops to function with each other.

**FIGURE 2:**
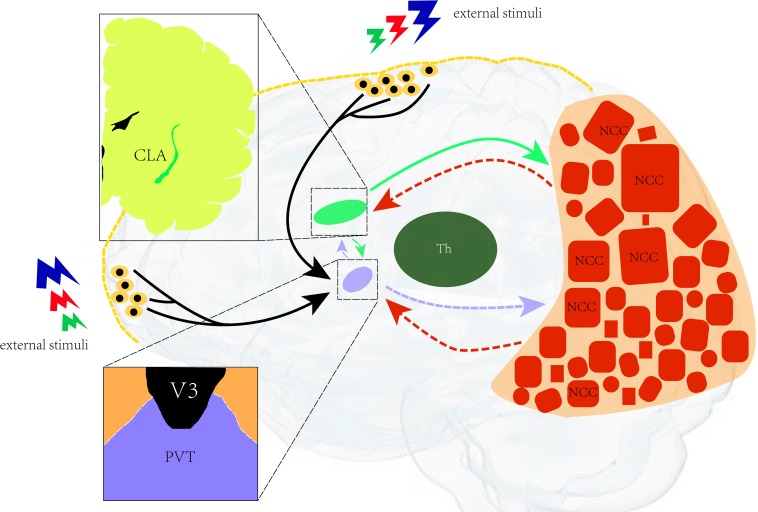
Illustration of the neural network for consciousness. A neural network of consciousness. The paraventricular nucleus is in control of arousal (closely related to the maintenance of consciousness) and the neurons in the posterior cerebral cortex. It is related to the integration of feelings and the generation of consciousness content. The claustrum is the key channel of the consciousness loop and the transmission of control information. CLA, claustrum; PVT, paraventricular; Th, thalamus; NCC, neural correlates of consciousness; V3, third ventricular.

Based on recent research, we hypothesize that there is a neural network of consciousness in which the paraventricular nucleus formally serves as the control nucleus of arousal, which is closely related to the maintenance of consciousness, and the neurons in the posterior cerebral cortex. It is related to the integration of feelings and the generation of consciousness content. Besides, the claustrum also represents the key channel of the consciousness loop and the transmission of control information (Figure 2).

## Conclusion

At present, consciousness is a very vague concept that lacks a specific and accurate definition. It is now widely accepted that there are two categories of consciousness: (1) content-related consciousness (i.e., the local state) and (2) the awakening state (i.e., the global state) (Crick and Koch, 2003, 2005; Tononi, 2012; Tononi and Koch, 2015). Although we need to establish a definition of consciousness, we should not be confined by the lack of definition. The cortex of each part of the brain plays an important role in the production of consciousness, especially the prefrontal and posterior occipital cortices and the claustrum. From this review, we are more inclined to believe that consciousness does not originate from a single brain section; instead, we believe that it originates globally.

The exploration of the intrinsic neurobiological mechanism of consciousness is of great significance. According to the latest research on consciousness, the paraventricular nucleus plays an important role in awakening, and the claustrum may represent the nucleus that controls information transmission and regulates the generation of consciousness. The aspects involved in consciousness include the level of consciousness and also the content of consciousness. In the past, consciousness was thought to emanate from the frontal hemispheres of the brain, but current research has found that the content of consciousness mainly originates from the hindbrain. According to the GW theory and the IIT, awareness research requires a large neural network. In order to understand the neurobiological mechanisms of consciousness, the generation of consciousness needs to link all of the critical nucleus functions and the cerebral cortex of the essential brain parts. Consciousness is not split, but instead the overall effects of consciousness are critical.

## Author Contributions

TZ wrote the manuscript. YZ revised the manuscript. HT proposed the idea for the manuscript. RX created the figures. JZ was responsible for checking the manuscript. JHZ was responsible for checking the whole manuscript as well.

## Conflict of Interest Statement

The authors declare that the research was conducted in the absence of any commercial or financial relationships that could be construed as a potential conflict of interest.
